# Do limbal relaxing incisions during cataract surgery still have a role?

**DOI:** 10.1186/s12886-022-02327-9

**Published:** 2022-03-04

**Authors:** Mohammad Saleh Abu-Ain, Motasem Mohammad Al-latayfeh, Mohammad Irfan Khan

**Affiliations:** 1 Prince Hamzah Hospital, Amman, Jordan; 2 Department of Special and General Surgery, The Hashemite University, Zarqa, Jordan; 3 Doncaster and Bassetlaw Teaching Hospitals NHS Foundation Trust, Armthorpe Road, Doncaster, UK

**Keywords:** Cataract surgery, Phacoemulsification, Astigmatism, Limbal relaxing incisions, Toric IOL

## Abstract

**Background:**

Though Limbal Relaxing Incisions (LRI) were used widely to correct pre-existing corneal astigmatism during cataract surgery, they have been replaced recently with the more expensive methods like the use of toric Intra Ocular Lenses (IOL) and femtosecond during cataract surgery. We conducted our study to re-evaluate the role of (LRI) in correcting pre-existing moderate corneal astigmatism during cataract surgery in settings where other options are neither available nor affordable.

**Methods:**

Retrospective analysis of all consecutive cases of LRI performed by a single surgeon at the time of cataract surgery to correct moderate corneal astigmatism (1.5-3D) in a community hospital over a period of 6 months. Corneal astigmatism, uncorrected distance visual acuity (UDVA) and best corrected distance visual acuity (CDVA) were recorded pre-operatively, 4 weeks and 3 months post-operatively. Data on age, intraocular lens (IOL) power, predictive refraction and post-operative spherical equivalent was also collected and analyzed. The number and position of LRI was determined based on the pre-existing corneal astigmatism using online calculator.

**Results:**

29 eyes of 25 patients with the mean age of 73.6 years (range: 46 to 90 years) and corneal astigmatism between 1.5 to 3D were included. Statistically significant reduction in the mean corneal astigmatism was recorded from 2.05 ± 0.45D preoperatively to 0.85 ± 0.56D postoperatively (*P* < 0.0001). All eyes showed reduction in astigmatism; 83% of eyes had < 1.0D post-operatively and 66% of eyes had < 0.75D. UDVA of 6/9 or better was recorded in 80% of eyes post-operatively (CDVA of 6/9 or better in 100%). The spherical equivalent was within 1.0D of the predictive refraction postoperatively in nearly all eyes (97%) and within 0.5D in 86% of the eyes. There were no peri-operative or post-operative complications were recorded in any case.

**Conclusion:**

Combining LRI and cataract surgery to address moderate degrees of corneal astigmatism is a safe, reliable and predictable option especially in areas where more expensive methods such as toric IOL or excimer laser are not available or affordable. LRI has no significant effect on the spherical equivalent and is an excellent tool in reducing patient’s spectacle dependence.

## Introduction

Cataract surgery has been established as one of the most commonly performed surgeries in the world. With improvements in intraocular lens (IOL) calculations, phacoemulsification techniques and with advances in available IOLs, patients’ expectations and demands are continuously on the rise. This has led to an ongoing drive pushing cataract surgeons to attain patients’ satisfaction and meet their expectations in having excellent postoperative unaided vision and being glasses independent. Achieving a predictable and repeatable postoperative spherical correction has largely been solved by the developments in ocular biometry and formulas. This has in turn led to emphasis on the reduction or elimination of pre-existing corneal astigmatism as this is the second most important factor to reduce spectacle dependence. It is estimated that corneal astigmatism of more than 1.0D is found in up to 40% [1] of patients presenting for cataract surgery, 1.5D or more is present in over 20% [2] and above 2.0D in 10% [3] of patients. There are various ways of correcting corneal astigmatism at the time of cataract surgery, and it includes incision placed on the steep axis of the cornea [4], addition of opposite clear corneal incision (OCCI) [5], single or paired limbal relaxing incisions (LRI) [4, 6, 7]and the use of toric IOLs [4, 6–9]. There are various published studies comparing toric IOLs vs LRI showing comparable results [7] while others showing toric IOLs to be superior and long lasting in achieving spectacle independence [6]. In this study we look at the use of LRI in reducing corneal astigmatism and improving outcomes in settings where toric IOLs are either not available or deemed too expensive.

## Patients and methods

This study is a retrospective analysis of all consecutive cases of LRI performed by a single surgeon over a period of 6 months. The study protocol has been approved by the Institutional Review Board (IRB) of the Hashemite University. The study protocol insured full patient data confidentiality and complied with Declaration of Helsinki. No informed consent was required by IRB since this is a retrospective study.

Patients presenting for cataract surgery with pre-existing corneal astigmatism between 1.5 and 3.0D were included in the study. Any patients with corneal astigmatism of less than 1.5D had on-axis incision alone or in combination with OCCI to reduce corneal astigmatism. On the other hand, patients presenting with more than 3D of corneal astigmatism were still offered LRI to reduce astigmatism but glasses independence was deemed unlikely to achieve and were not included in this study. 29 eyes of 25 patients were identified and included in this study and all patients had detailed history and complete ophthalmic examination including full pre-operative cataract assessment. All patients had normal adnexa and anterior segment examination, normal IOP and nearly normal fundus examination with no previous lid or ocular surgery with corneal astigmatism between 1.50 to 3.0 D. We considered any ongoing chronic ocular pathology or corneal ectasia as an exclusion criterion for the purpose of this study.

The medical records of those patients were identified from theatre records and evaluated. Data collection included uncorrected distance visual acuity (UDVA), best corrected distance visual acuity (CDVA) and corneal astigmatism, pre and post operatively. Data on age, gender, IOL power, predicted spherical refraction and post-operative spherical equivalent was also collected. Patients were identified using partial coherence interferometry IOLMaster (Carl Zeiss Meditec, Germany), of having corneal astigmatism between 1.5–3.0D and had corneal topography to confirm the degree and axis of the astigmatism using Pentacam Eye Scanner (Oculus Optikgeraete GmbH; Wetzlar, Germany) as well as to rule out any irregular astigmatism or subtle corneal ectasia which were both considered as contra-indications for the LRI.

Patients with corneal astigmatism of between 1.5–2.0D had their main phaco incision (Clear corneal incision of 2.8 mm) on the steep meridian with one LRI at 180 degrees opposite to the main incision. Patients with more than 2.0D of corneal astigmatism had their two LRI on the steep meridian 180 degrees apart. The calculations of the length and position of LRI were done using the Donnenfeld nomogram with the online calculator; http://www.lricalculator.com.

### Procedure

**Fig. 1:**
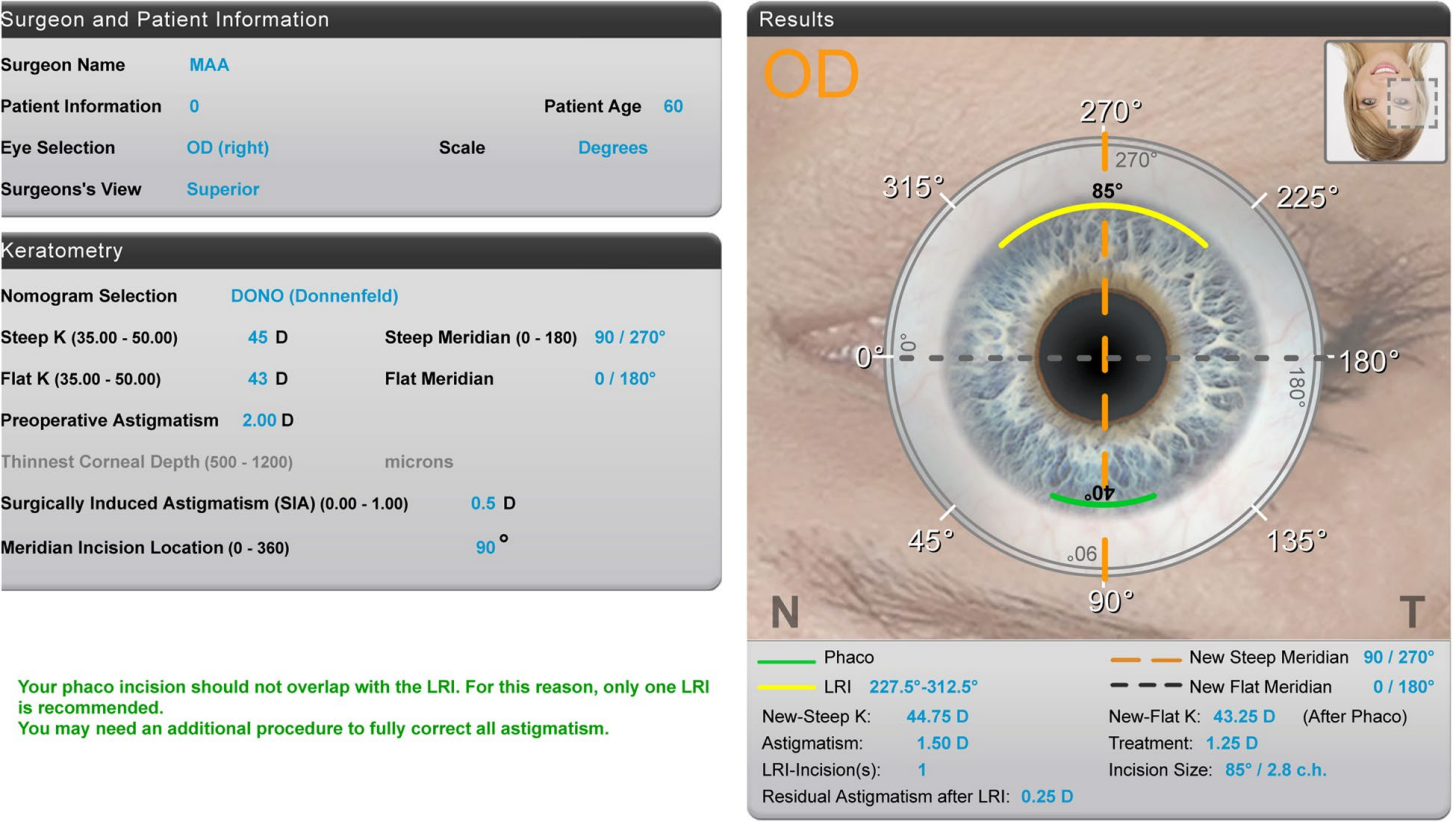
LRI online calculation showing an example with 2.0D of corneal astigmatism having the steep meridian at 90 degrees and the main phaco incision at the steep meridian with SIA of 0.5D. The recommendation was for one LRI at 270 degrees with 85 degrees size using a guarded diamond knife having a pre-set depth of 550 μm. The residual corneal astigmatism was predicted be 0.25D

All procedures were performed by the same experienced surgeon using standard phacoemulsification under topical anesthesia. All patients had immediate pre-operative limbal-corneal marking while sitting upright and looking straight ahead using the slit lamp to avoid any cyclotorsion of the eyeball which could be induced by lying supine as per previously published technique [10]. All patients were fully consented and informed about the expectations along with potential complications. Insulin needle was used to make a linear radial corneal abrasion at the limbus after installing topical anesthesia with fluorescein (Proxymetacaine HCL 0.5% & Flourescein 0.25% minims) preoperatively. On the operating table, the site and the length (in clock hours to avoid any possible effect of corneal diameter) of the LRI were marked using Mendez gauge (manufactured by Duckworth and Kent, England, UK) after identifying the steep corneal meridian with the aid of pre-placed corneal reference marks. Single or paired LRI were performed prior to phacoemulsification procedure using a 550 μm diamond guarded blade and placed at least 0.5 mm anterior to the limbus. All patients had 2.8 mm clear corneal incision as the main phacoemulsification incision with no wound enlargement. Surgical induced astigmatism for the surgeon was previously calculated to be 0.5D which was used for the online LRI calculator (Fig. 1) and all patients had Akreos AO60 (Bausch & Lomb) IOL in the bag.

## Results

**Fig. 2:**
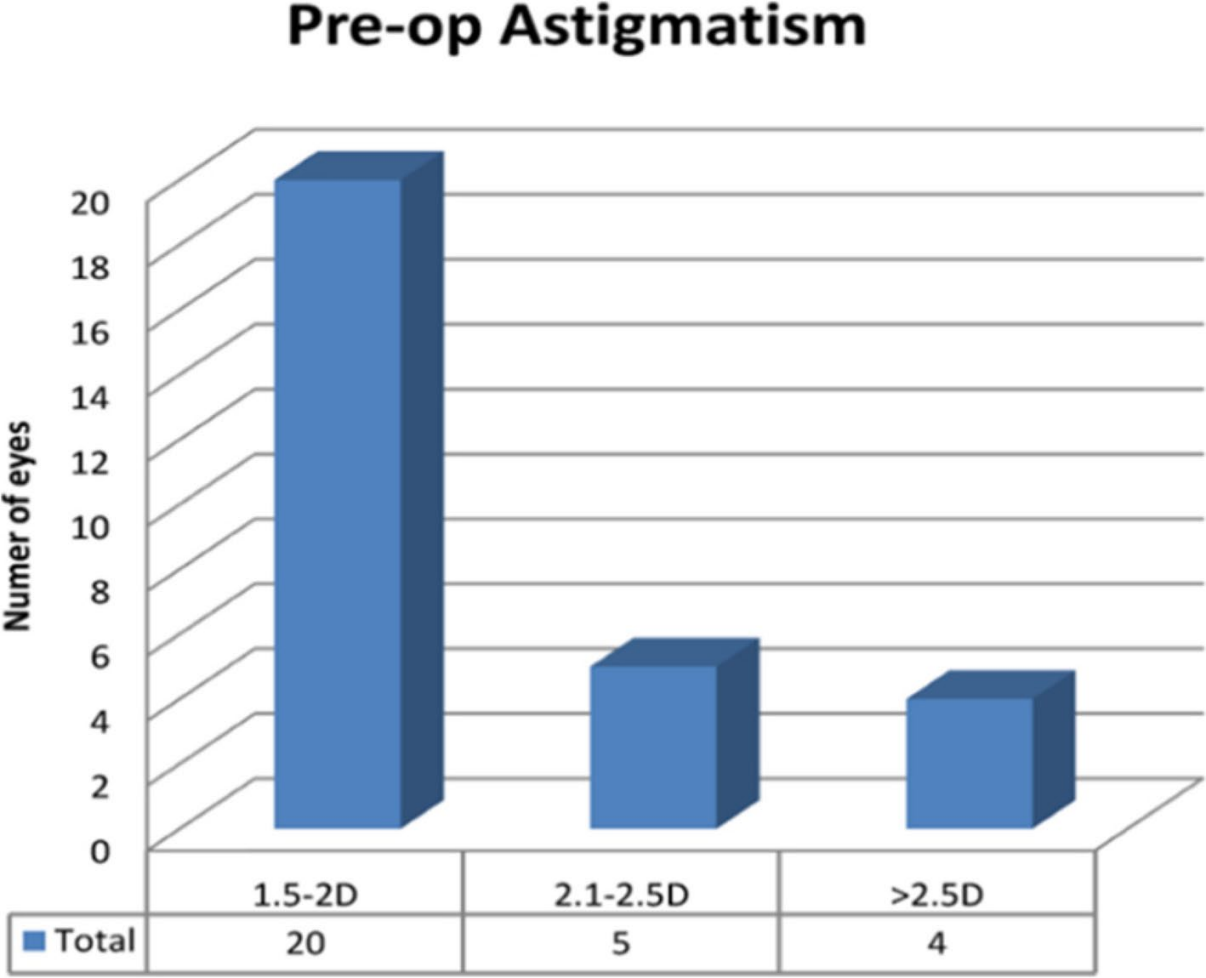
Distribution of pre-operative astigmatism

**Fig. 3:**
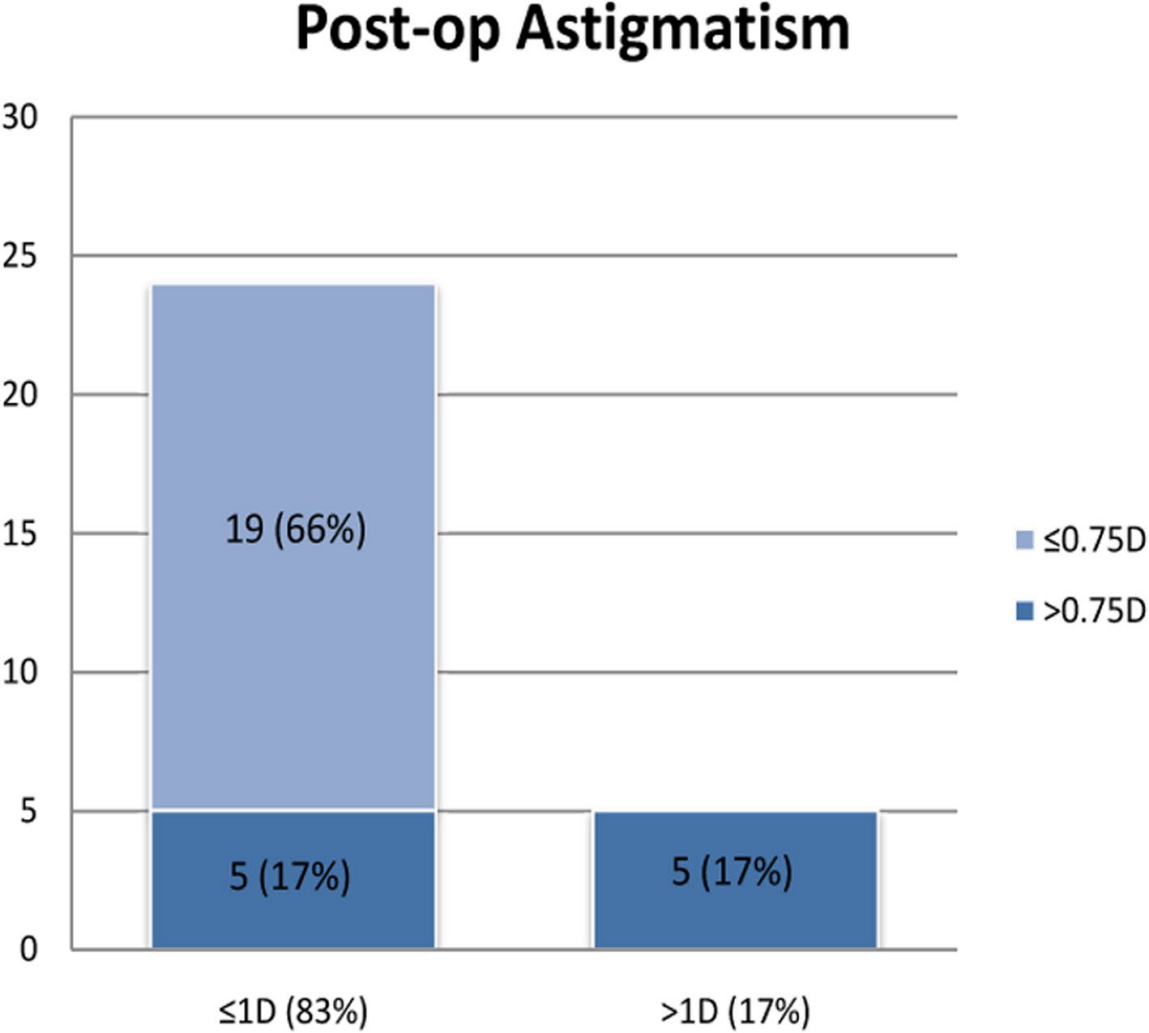
Distribution of post-operative astigmatism

**Fig. 4:**
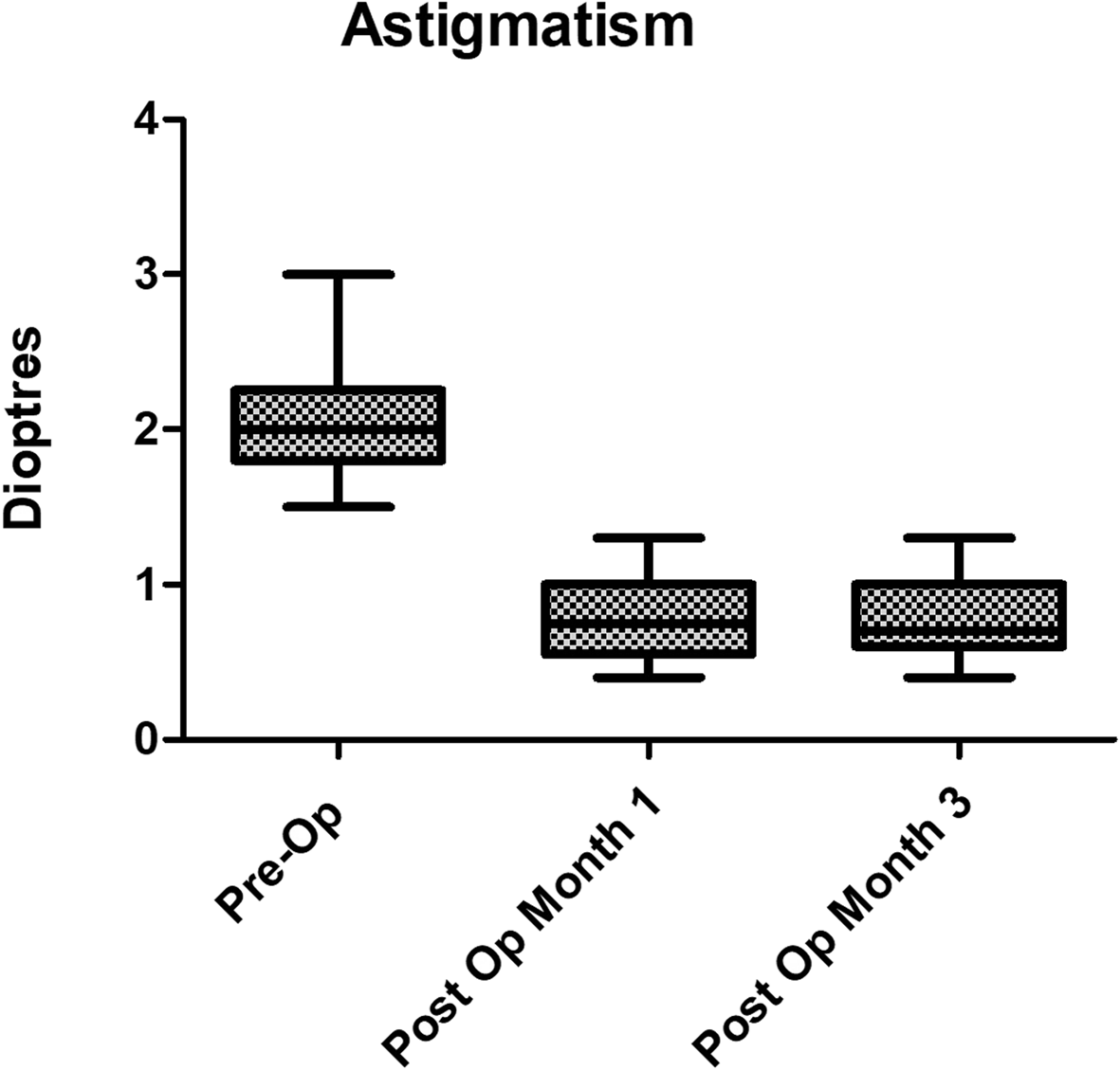
Corneal astigmatism preoperatively (pre-Op) and post operatively at month 1 and month 3 showing the range and mean values

**Fig. 5:**
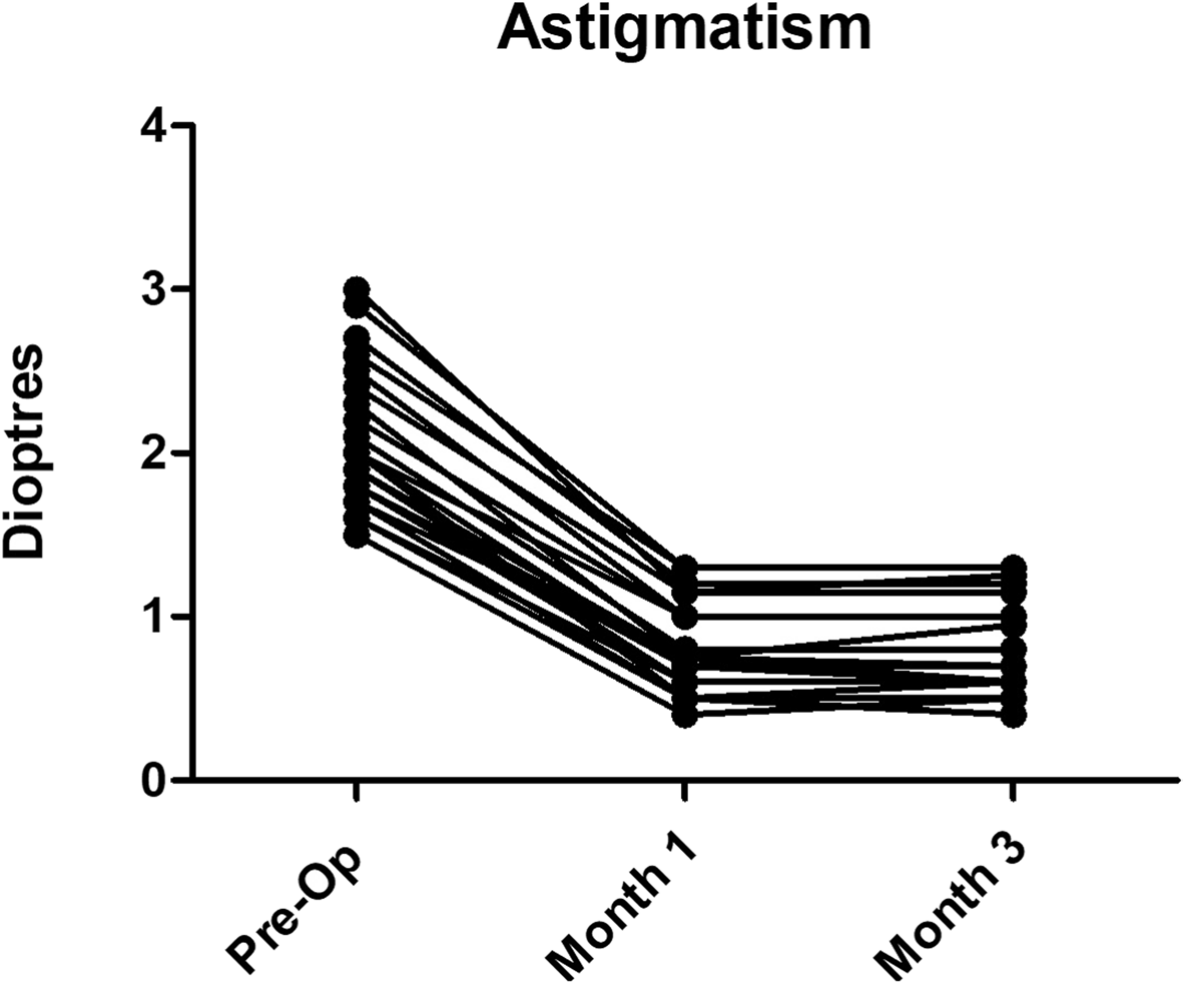
Corneal astigmatism showing statistically significant improvement post operatively with no significant difference between month 1 and month 3 results

Twenty-nine eyes of 25 patients were included in the study (15 right and 14 left eyes while 12 male and 13 female patients) with the mean age of 73.6 years (range: 46 to 90 years). All procedures were uneventful, with no intra-operative or post-operative complications. Patients were reviewed at 1 month and 3 months post-operatively. UDVA and CDVA were reported in addition to complete ophthalmic examination including subjective refraction. Corneal topography was also performed at 3 months postoperatively to ensure stability of cornea has been achieved. Mean pre-operative corneal astigmatism was 2.05D ± 0.45 (Range: 1.5-3D) distributed as: 1.5–2.0D in 20 eyes (69%), 2.1–2.5D in 5 eyes (17%) and > 2.5D in 4 eyes (14%) as shown in Fig. 2. Post-operative astigmatism was < 1.0D in 24 eyes (83%), < 0.75D in 19 eyes (66%) and between 1.0–1.25D in 5 eyes (17%) as shown in Fig. 3. All eyes had reduction in their corneal astigmatism and UDVA was 6/9 or better in 80% of eyes (23 eyes) which increased to 100% with correction in eyes with no co-morbidities. Post-operatively 97% eyes were within 1.0D of the predicted spherical equivalent and 86% of eyes were within 0.5D. A statistically significant reduction in the mean corneal astigmatism was noticed from 2.05D ± 0.45 pre-operatively to 0.85D ± 0.56 at 3 months’ post-operatively (*p* < 0.0001). The improvement in corneal astigmatism was maintained between 1 and 3-month’s post operatively as shown in Fig. 4 and the journey of individual cases is highlighted in Fig. 5 which shows that all eyes had improvement in their corneal astigmatism. Table 1 also shows the pre-operative and post-operative parameters with no statistically significant difference between the results at one month versus 3 months postoperatively. Statistical analyses were performed using Graph Pad Prism software with all statistical analysis being 2 sided and values less than 0.05 were considered significant.

**Table 1:** Comparison and pre and post-operative parameters; UDVA = Uncorrected distance visual acuity in LogMAR. CDVA = corrected distance visual acuity in LogMAR, D = Diopters, SD = standard deviation

Parameter	Pre-Op (Mean ± SD)	1 Month	***P*** Value	3 Months	***P*** Value
**UDVA (LogMAR)**					
Mean ± SD	0.61 ± 0.19	0.16 ± 0.14	<.0001	0.18 ± 0.12	<.0001
Range	0.4–1	0.10–0.03		0.11–0.02	
**CDVA (LogMAR)**					
Mean ± SD	0.42 ± 0.14	0.007 ± 0.06	<.0001	0.006 ± 0.07	<.0001
Range	0.2–0.8	−0.1 – 0.2		−0.1 – 0.24	
**Corneal Astigmatism (D)**					
Mean ± SD	2.05 ± 0.45	0.79 ± 0.27	<.0001	0.85 ± 0.56	<.0001
Range	1.5–3	0.4–1.3		0.4–1.2	

## Discussion

It is well established that addressing corneal astigmatism at the time of cataract surgery can increase spectacle independence. This in turn will not only have practical advantages for the patient but in addition will provide cosmetic and economic benefits [9]. When astigmatism is corrected with glasses, it creates meridional magnification which can produce asymmetric and distorted retinal images. These images can in turn reduce spatial perception [11] and prove to be quite challenging for the elderly population [12] undergoing cataract operation. However, if corneal astigmatism is corrected at corneal plane or at the IOL plane, the above-mentioned challenges could be avoided [13].

We also know that placing clear corneal incision on the steep axis results in flattening of cornea in that meridian [5] as clear corneal incisions and astigmatism has been extensively examined. The longer the incision or closer the incision to the center of the cornea, the greater is its flattening effect [14]. There are various studies in the literature that compares LRI against toric IOLs with very mixed results; in some studies, LRIs have shown comparable results to toric IOLs [8] while in other studies toric IOLs have been found to be more efficacious [9, 15]. However, toric IOLs are more costly and may require additional surgical procedure to re-align the IOL. LRIs are cheaper to perform and may still be an effective option in settings where either toric IOLs are not available or deemed too expensive.

During cataract surgery, corneal astigmatism of < 1.5 D can be easily managed by smart planning. Placement of the clear corneal incision on the steep meridian for example would help in reducing astigmatism and its effect would depend on the location, width and structure of the incision [16]. This basic technique would be enough for addressing corneal astigmatism of up to 1.0D and the residual corneal astigmatism of < 0.5D could still be sufficient for being glasses independent [17]. For patients having corneal astigmatism between 1.0 and 1.5D, the use of opposite clear corneal incision (OCCI) is an added option. Paired OCCIs have been found to be a reliable, effective and predictable solution in enhancing the effect of a single clear corneal incision in addressing corneal astigmatism at the time of cataract surgery [5]. The above-mentioned options would not require any additional equipment and are simple and basic methods to improving preexisting corneal astigmatism and have been reported to address up to 2.0D of astigmatism [18] depending on the size of incision. Steeper corneas could be approached by performing LRI at time of cataract surgery [2, 4, 19, 20] when toric IOLs are not an available option. The procedure is easy to perform and could be safely adapted by most cataract surgeons without adding any significant operating time.

In this study we included patients who had pre-existing corneal astigmatism between 1.5 and 3D. These patients desired reduced spectacle dependence where the option of toric IOL was neither available nor affordable. Patients who had less than 1.0D astigmatism had on-axis corneal incision (with or without enlargement of wound depending on the magnitude of astigmatism) while OCCI was added for cases where astigmatism was between 1.0 and 1.5D. All 29 eyes included underwent the procedure of LRI at the start of operation without having any intra-operative or post-operative complications either due to LRI or cataract surgery. Mean pre-operative corneal astigmatism in our study was reduced from 2.05D ± 0.45 to 0.85D ± 0.56 post-operatively (*p* < 0.0001) which compares well with the published literature [16]. All eyes showed improvement in corneal astigmatism magnitude; 100% of eyes had more than 1.5D of corneal astigmatism pre-operatively but none of the eyes had more than 1.25D of residual astigmatism post-operatively. These results are quite encouraging and in keeping with most published data [4, 19, 20] and our study also showed no statistically significant difference between the results at 1 and 3 months post-operatively (p 0.8825). In our series, 83% eyes had less than 1.0D residual astigmatism post-operatively and 17% (5 eyes) had residual astigmatism between 1.0 and 1.25D but all these eyes had high pre-operative astigmatism (> 2.4D).

LRI has also been called peripheral corneal relaxing incision (PCRI) in more recent literature [21] as this term describes the procedure more accurately. There are various ways of calculating and planning the location and length of LRI which is made quite easy by the website: www.lricalculator.com. The incision used for cataract surgery can be made use of or it can be made temporally in all surgeries accompanied by paired LRIs on the steep meridian. However, the calculator would adjust the length of the LRIs to compensate for the SIA. The calculator can be used with clock hours or degrees as per preference of the surgeon and in good hands it has proven to be very effective, predictable, safe and cost-effective technique for addressing corneal astigmatism.

LRI has also been used safely with multifocal IOL [22] with minimal effect on the final post-operative spherical equivalent necessitating no change in the IOL calculations [23]. It also has the advantage of keeping the central cornea intact for possible excimer laser surgery if the post-operative astigmatic results were sub-optimal [24]. By selecting appropriate patients, performing detailed assessment including corneal topography, the risk to patient is minimized as well as conditions like corneal ectasia are excluded where LRI would be contraindicated due to irregular nature of astigmatism.

Although toric IOLs have been around for some time, they may still not be available in certain practices or countries. As an example, even in United Kingdom’s state-owned National Health Service (NHS), toric IOLs are not available routinely to patients in all parts of the country. There are some hospitals where toric IOL is offered to the patients however there are still significant hospitals including tertiary units where toric IOL is not an option on the NHS. Moreover, there are certain hospitals where toric IOL is only offered if patients have corneal astigmatism above an agreed cut off which could be 2 or 3 diopters. Similarly, there are countries like Jordan where toric IOL or laser refractive options for correcting corneal astigmatism may not be available due to cost/affordability and LRI would be a cheaper way to provide some improvement in patient’s UDVA or spectacle dependence. As a rough guide, in UK the cost of toric IOL is around 8–10 times higher than the price of LRI blade. In UK this will amount to at least around 80 pounds difference in the cost; which could be higher if toric IOL rotates and patient is taken back to theatre. Hence, we believe the use of LRI is an efficient and significantly cheaper alternate to the use of toric IOL in offering patients similar benefits without the attached price tag.

The limitations of this study could be the retrospective nature of the cohort and the relatively smaller sample. This study also included eyes with corneal astigmatism less than 3D, which may have implications on generalizing these results for higher degree of astigmatism.

## Conclusion

Combined LRI and phacoemulsification appears to be safe and effective to correct moderate degrees of corneal astigmatism during cataract surgery especially in areas where more expensive method like toric IOL is unavailable or unaffordable. LRI has no significant effect on the spherical outcome and has an excellent safety profile. We believe it still has a role and cataract surgeons should consider it as an option in helping patients enjoy spectacle independence where modern techniques might not be available or affordable. This study helps to re-iterate the confidence in the use of LRI and its success in improving patient’s spectacle dependence even in settings with limitations.

## Data Availability

Data analyzed during this study are included in this published article and detailed data are available upon request from the corresponding author (MSA).
